# Protein–Protein Interactions with Connexin 43: Regulation and Function

**DOI:** 10.3390/ijms19051428

**Published:** 2018-05-10

**Authors:** Paul L. Sorgen, Andrew J. Trease, Gaelle Spagnol, Mario Delmar, Morten S. Nielsen

**Affiliations:** 1 Department of Biochemistry and Molecular Biology, University of Nebraska Medical Center, Omaha, NE 68198, USA; psorgen@unmc.edu (P.L.S.); andrew.trease@unmc.edu (A.J.T.); gspagnol@unmc.edu (G.S.); 2 Leon H Charney Division of Cardiology, NYU School of Medicine, New York, NY 10016, USA; Mario.Delmar@nyumc.org; 3 Department of Biomedical Sciences, Faculty of Health and Medical Sciences, University of Copenhagen, DK-2200 Copenhagen, Denmark

**Keywords:** gap junction, connexin, protein–protein interaction, intrinsically disordered protein, post-translational modification, intercalated disc

## Abstract

Connexins are integral membrane building blocks that form gap junctions, enabling direct cytoplasmic exchange of ions and low-molecular-mass metabolites between adjacent cells. In the heart, gap junctions mediate the propagation of cardiac action potentials and the maintenance of a regular beating rhythm. A number of connexin interacting proteins have been described and are known gap junction regulators either through direct effects (e.g., kinases) or the formation of larger multifunctional complexes (e.g., cytoskeleton scaffold proteins). Most connexin partners can be categorized as either proteins promoting coupling by stimulating forward trafficking and channel opening or inhibiting coupling by inducing channel closure, internalization, and degradation. While some interactions have only been implied through co-localization using immunohistochemistry, others have been confirmed by biophysical methods that allow detection of a direct interaction. Our understanding of these interactions is, by far, most well developed for connexin 43 (Cx43) and the scope of this review is to summarize our current knowledge of their functional and regulatory roles. The significance of these interactions is further exemplified by demonstrating their importance at the intercalated disc, a major hub for Cx43 regulation and Cx43 mediated effects.

## 1. Introduction

The Cx43 carboxyl terminal (Cx43CT) domain plays a role in the trafficking, localization, and turnover of gap junction channels via numerous post-translational modifications and protein–protein interactions [1,2,3,4,5]. The Cx43CT is also important for regulating junctional conductance and voltage sensitivity [6,7,8,9]. Structural studies from our laboratory revealed that the Cx43CT as well as the CT domain from other connexins are predominately unstructured [10,11,12,13]. Intrinsically disordered domains are now well recognized to be loci for regulation of protein function because their conformation can be readily modulated by the local environment, phosphorylation, and interaction with proteins and small-molecules. We and others have shown that the Cx43CT binds multiple proteins, some of which have been shown to modulate channel function (for review see [14]). These data strongly suggest that protein–protein interactions mediated by any part of the CT are likely to have regulatory effects. Numerous excellent reviews have summarized the functional significance of these Cx43-interacting proteins [15,16,17]; here we provide a different perspective. We separated the proteins known to affect Cx43 function into three categories. The first are those proteins that directly interact with the CT and are associated with trafficking Cx43 to the gap junction plaque and open gap junction channels. Cx43-protein interactions identified from cell biology studies (e.g., immunoprecipitation and co-localization) that have been confirmed using different biophysical techniques (e.g., nuclear magnetic resonance, X-ray crystallography, and surface plasmon resonance) are considered a “direct” interaction. The second are those proteins that directly interact with the CT and are associated with channel closure, disassembly, and degradation. The third, which will not be a focus of this review, are those proteins that can affect all aspects of the Cx43 life cycle, but no evidence exists they directly interact with the Cx43CT (Table 1; albeit we realize a number of the proteins in Table 1 will eventually be shown to directly interact with Cx43 or may never be identified because binding requires a connexin embedded within the membrane or in context of a connexon, thus posing extreme challenges to performing in vitro assays). Additionally, we will not focus on those post-translational modifications such as ubiquitination, sumoylation, methylation, phosphorylation, and hydroxylation that form covalent bonds with connexins to modify function (for review see [18]). For the proteins that directly interact, we provide their location on the Cx43CT domain, residues (de)phosphorylated where necessary, and their diameter as estimated from their molecular weight (Available online: http://www.calctool.org/CALC/prof/bio/protein_size). Of note, these values are on the conservative side because proteins like ZO-1 and 14-3-3 have multiple modular domains and would have a larger diameter. For the Cx43CT, we combined the knowledge that the intrinsically disordered Cx43CT domain (length of 3.8 Å per residue; [19]) can contain as high as 35% α-helical structure (length of 1.50 Å per residue) depending on the level of phosphorylation [20]. The rationale for this perspective is to visually illustrate that only a small number of proteins can bind at any one time. The importance of Cx43 cellular localization (spatial), Cx43CT phosphorylation state, as well as the cellular condition (temporal) will help determine which proteins will bind the Cx43CT domain.

## 2. Direct Interactions with Cx43 and Their Functional Consequence

### 2.1. Interactions that Promote Synthesis, Trafficking to the Gap Junction Plaque, and Channel Opening

**Figure 1:**
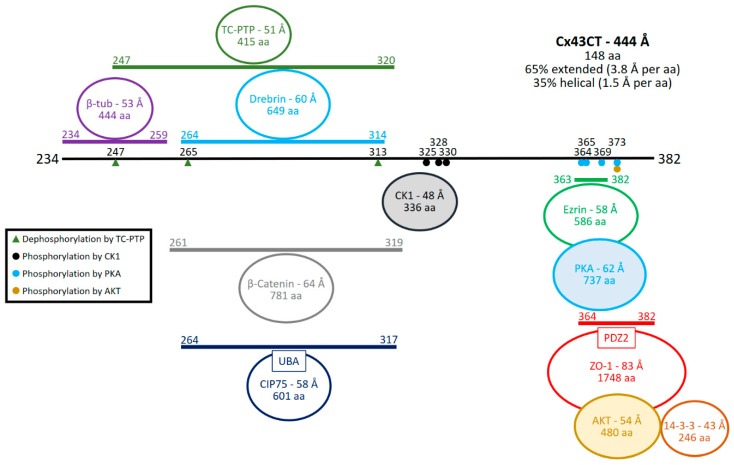
Protein partners that directly interact with the Cx43CT domain to promote intercellular communication. The black line represents Cx43CT domain residues 234–382. Provided for each Cx43CT protein partner (circle) is its diameter (in Å) as estimated from their molecular weight, and number of amino acids (aa), and the Cx43CT residues affected as a result of the interaction (lines). If the protein partner is a kinase or phosphatase, the Cx43CT residues affected are labeled on the Cx43CT (circle or triangle). Abbreviations are as follows: β-tubulin (β-tub), T-cell protein tyrosine phosphatase (TC-PTP), Connexin interacting protein 75 kDa (CIP75), Ubiquitin-associating domain (UBA), Casein kinase 1 (CK1), Protein kinase A (PKA), Zonula Occludens 1 (ZO-1), and Protein kinase B (AKT). Kinases have been highlighted (shaded circle).

Intercellular coupling is eventually determined by the number of open channels in gap junction plaques, which is governed by the synthesis, forward trafficking, and channel open probability. A number of protein partners affect these processes (Figure 1).

Cx43 is translationally integrated into the endoplasmic reticulum (ER) and oligomerization occurs only after exit of the ER in the trans-Golgi network [71]. One of the first proteins likely to directly interact with Cx43 is the Connexin Interacting Protein of 75 kDa (CIP75). CIP75 interacts with Cx43CT residues K264-Q317 through its ubiquitin-associated (UBA) domain [72,73]. The importance of CIP75 is to mediate ER associated degradation of Cx43 for quality control and fine-tune the level of expression through dislocation of Cx43 from the ER and proteasomal degradation [73,74,75,76]. Use of cellular denaturants increased the association of CIP75 with Cx43, suggesting only pools of Cx43 lacking association with CIP75 escape ER dislocation and travel to the Golgi [75]. Upon exiting the trans-Golgi network, Cx43 containing vesicles are transported via the microtubular network to the plasma membrane [77].

Microtubular transport of connexons coincides with the recruitment of a number of protein interactors to the Cx43CT, a number of which have been implicated, however a direct interaction was not confirmed (Table 1; for review see [4,78,79]). In addition to microtubules, the actin cytoskeleton aids in connexon delivery to the gap junction plaque (for review see [80]). Curiously, regulation of Cx43 forward trafficking may in part be regulated by internally translated fragments of the Cx43CT [81]. One of these fragments, GJA1-20k, was recently shown to stabilize filamentous actin and suggested to help target microtubules to cell–cell junctions [82]. Full length Cx43 did not stabilize actin and the relation between the ability of GJA1-20k and Cx43 (see below) to target microtubules to the membrane remains to be established.

In proximity of the plasma membrane, the actin- and protein kinase A (PKA)-binding protein Ezrin, binds the Cx43CT and enables PKA to phosphorylate Cx43CT serine residues. In particular, phosphorylation of S364 is a likely precursor to binding with the tight junction protein Zonula occludens 1 (ZO-1), another actin scaffolding protein [83]. Functional studies demonstrating increased gap junction intercellular communication following activation of PKA support this hypothesis [84,85]. Work by Pidoux et al. 2014, identified the minimal binding motif of Cx43CT for Ezrin as ^366^RASSR^370^ using a peptide screening approach [86]. Furthermore, PKA and ZO-1 interact with the Cx43CT over the same region as Ezrin (S364-I382), however phosphorylation by PKA (S365, S369) did not appear to alter binding of Ezrin to Cx43, nor binding of ZO-1 [86,87]. Work from Thévenin et al. 2017, and others have highlighted phosphorylation of S373 as a critical modulator of ZO-1 binding, a site phosphorylated by both PKA and protein kinase B (AKT) [83,87,88,89]. Association with ZO-1 is a critical mediator of gap junction plaque size; when bound to ZO-1 Cx43 is retained in the perinexal region “poised” for docking with apposing connexons, and upon release Cx43 is incorporated into the gap junction plaque proper [88,89,90,91]. Whether Ezrin and ZO-1 simultaneously bind the Cx43CT remains to be determined, but based on their size and location of binding on the Cx43CT, it seems unlikely.

Capture and incorporation of Cx43 containing vesicles at the plasma membrane (gap junction periphery) has been attributed to 14-3-3 [92,93,94]. Like Ezrin, 14-3-3 interacts with the Cx43CT in the same region as ZO-1, hovering over S373 [94]. Unlike the reduced binding of ZO-1, phosphorylation of S373 by PKA enhances 14-3-3 binding and likely serves as a switch of perinexal Cx43 to junctional Cx43 through tethering to integrins (specifically integrin α5; [88,89,94]). Taken together these studies highlight the intricacy of spatial-temporal and post-translational regulation of Cx43 trafficking to the gap junction plaque and suggest that association of Ezrin (and PKA) with the Cx43CT precedes association with ZO-1. This is further advanced by phosphorylation of S373 promoting the exchange of ZO-1 for 14-3-3 and incorporation into the gap junction plaque [88,89,94]. Of note AKT and 14-3-3 proteins are also involved in gap junction disassembly, a topic covered in the next section. Once incorporated into the plaque a number of interactions serve to stabilize and maintain Cx43 and control channel maturation (opening; for review see [95]).

Fully open channels require phosphorylation by casein kinase 1 (CK1) on residues S325, S328, S330 [96]. Interestingly, Cx43 knock-in mice in which Cx43CT residues S325, S328, and S330 were replaced with glutamic acids (phospho-mimicking) were immune to acute and chronic pathological gap junction remodeling and ventricular arrhythmias after transverse aortic constriction [97]. In addition to channel opening, stability of the gap junction plaque regulates gap junction intercellular communication. Direct protein interaction with microtubules via β-tubulin and association with the actin cytoskeleton through the scaffolding protein Developmentally Regulated Brain Protein 1 (Drebrin) are two key interactions, which stabilize gap junctions (for review see [98,99]). β-tubulin binds the Cx43CT over Y247, a known site of phosphorylation by Src kinase, and Drebrin binds over Y265 and Y313, two other substrates for Src phosphorylation [100,101,102,103,104,105,106]. Importantly, the interaction of β-tubulin with the Cx43CT likely occurs subsequent to plasma membrane incorporation as a direct interaction prior to plasma membrane incorporation would prevent Cx43 trafficking to the membrane (no motor proteins). This hypothesis is supported by data from Francis et al. 2011, indicating that Cx43 regulates microtubule dynamics at plasma membrane [107]. NMR and cell based work from our laboratory identified a phosphatase T-cell Protein Tyrosine Phosphatase (TC-PTP) which directly interacts with the Cx43CT and dephosphorylates the Y247 and Y265 reversing the down-regulating effects of Src kinase (described further in the next section; [108]).

Finally, β-catenin is another protein identified to interact with Cx43. In response to Wnt signaling, β-catenin can interact with the Cx43 gene to increase transcription as well as modulate gap junction stability at the plaque [109,110,111,112]. Works from several laboratories have shown indirect evidence of this interaction at the plaque by reciprocal co-immunoprecipitation as well as co-localization [109,113]. β-catenin was added in this section because we recently identified a direct interaction with the Cx43CT domain over three areas (residues G261-T275, S282-N295, and N302-R319) using a combination of surface plasmon resonance (SPR) and NMR experiments [114].

### 2.2. Interactions that Promote Channel Closure, Gap Junction Disassembly, Internalization and Degradation

**Figure 2:**
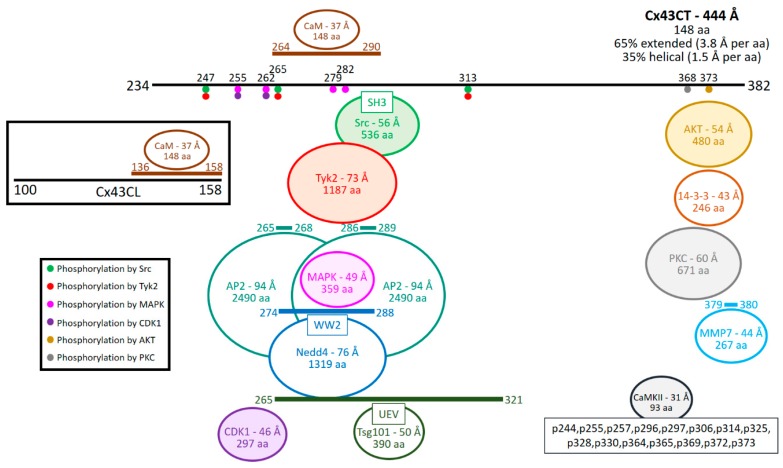
Protein partners that directly interact with the Cx43 CT and CL domains to impede intercellular communication. The black lines represents Cx43CT domain residues 234–382 and Cx43CL domain residues 100–158. Provided for each Cx43 CT and CL protein partner (circle) is its diameter (in Å) as estimated from their molecular weight, and number of amino acids (aa), and the Cx43CT residues affected as a result of the interaction (lines). If the protein partner is a kinase, the Cx43CT residues affected are labeled on the Cx43CT (circle). Abbreviations are as follows: Calmodulin (CaM), Src homology 3 domain (SH3), Tyrosine kinase 2 (Tyk2), Mitogen-activated protein kinase (MAPK), Neural precursor cell expressed developmentally down-regulated protein 4 (Nedd4), Cyclin-dependent kinase 1 (CDK1), Tumor susceptibility gene 101 protein (Tsg101), Ubiquitin E2 variant domain (UEV), Protein kinase B (AKT), Protein kinase C (PKC), matrix metalloproteinase-7 (MMP7), and Ca^2+^/calmodulin-dependent protein kinase II (CaMKII). Kinases have been highlighted (shaded circle).

Similarly, to facilitating coupling, down regulation of Cx43-mediated intercellular communication requires a number of direct protein interactions and phosphorylation events (Figure 2). Indeed, phosphorylation of Cx43 by Src is a key initiator of gap junction closure, internalization, and turnover [103,104,115,116,117,118,119]. Src-induced phosphorylation of Cx43 has been correlated with channel closure [101]. Current research suggests a “particle–receptor” mechanism for Src-mediated channel closure similar to that proposed for pH gating of Cx43 channels [7,104,120]. The impact of Src phosphorylation on channel activity is decreased electrical coupling by reducing open probability and changes in selectivity [121]. Work from our laboratory and others support an additional mechanism of Src to decrease gap junctional intercellular communication: the altering of Cx43 protein partners to enhance degradation. A commonality between the proteins that link Cx43 to the cytoskeleton is that Src can inhibit their interaction. For example, Cx43CT residues Y247 and Y265 phosphorylated by Src inhibit the binding of β-tubulin and Drebrin, respectively [122].

In the case of β-tubulin, at the gap junction plaque, this may be a mechanism in the disassembly process; at the trans-Golgi network, in cardiomyocytes this may re-route trafficking from the intercalated disc to lateral membranes; or inhibit trafficking to the plasma membrane altogether, leading to increased proteasomal and/or lysosomal degradation. For Drebrin, depletion in cells results in impaired cell–cell coupling, internalization of gap junctions, and targeting of Cx43 for degradation [123]. While phosphorylation of the Cx43CT by Src does not inhibit ZO-1 binding, we found that active c-Src can compete with Cx43 to directly bind ZO-1 [124]. Studies from the Gourdie and Lampe laboratories would suggest blocking these protein partners would transition Cx43 from the non-junctional plasma membrane into the gap junction plaque, and then through the degradation pathway(s) [91]. Finally, Src activation also indirectly leads to serine phosphorylation by AKT (S373), PKC (S368), and MAPK (S255, S279, and S282) that contributes to reduced Cx43 at the plasma membrane. AKT may act in a similar manner as Src in that phosphorylation of S373 inhibits the Cx43 interaction with ZO-1 [88]. In addition, phosphorylation of S373 enables the binding of 14-3-3 leading to gap junction ubiquitination, internalization, and degradation during acute cardiac ischemia [94]. Altogether, the data point to Src playing a significant role in inhibiting Cx43-mediated cell-to-cell communication by channel closure and enhanced degradation.

In addition to Src, another tyrosine kinase identified to directly interact with and phosphorylate the Cx43CT was the Janus kinase family member non-receptor tyrosine-protein kinase 2 (Tyk2; [125]). Interestingly, Tyk2 can functionally substitute for Src as work from our laboratory identified that it phosphorylates Cx43CT residues Y247 and Y265 and results in concomitant loss of coupling and disassembly of gap junction plaques [125]. While phosphorylation of these sites by either Tyk2 or Src would result in disruption of the direct binding of β-tubulin and Drebrin, one difference is that Tyk2 unlikely disrupts the Cx43/ZO-1 interaction as Tyk2 does not contain a SH3 domain (for review see [126]). Whether Tyk2 binds to Cx43 via its SH2 domain or FERM domain remains to be determined [127,128,129]. It is becoming clear that overlap in the phosphorylated residues of Cx43 by a number of kinases provides the cell with a highly dynamic ability to alter gap junction function in response to various initial stimuli. In addition, like Src, activation of Tyk2 coincides with increased phosphorylation of S279/282 by MAPK and S368 by PKC [125]. MAPK also phosphorylates Cx43 residues S255 and S262, all of which alter the secondary structure of the Cx43CT to increase α-helical content, a mechanism which can promote or inhibit interactions with other protein partners [20].

One protein partner that undergoes recruitment following MAPK activation, is the E3 ubiquitin ligase Neural precursor cell expressed developmentally down-regulated 4 (Nedd4; [130]). Specifically, work by Leykauf et al. 2006, demonstrated that phosphorylation of S279/282 increased the affinity (*K_D_* pS279/282 585 µM vs non-pS279/282 1064 µM) of Nedd4 for Cx43 [131]. Our laboratory confirmed this approximate 2-fold increase in the binding affinity for Nedd4 via NMR [132]. Furthermore, we determined that Nedd4 binds to the Cx43CT primarily through its WW2 domain via the PPXY motif (P283-Y286; [132]). Importantly, other proteins also interact with the Cx43CT in proximity to the PPXY motif, these are tumor susceptibility gene 101 (Tsg101) and the AP2 adaptor protein complex (AP2) both of which are involved in the endocytosis and retrograde trafficking of Cx43 [133,134]. In addition to MAPK, Src phosphorylation also primes Cx43 for phosphorylation by PKC at S368 [102]. A point worth noting is that phosphorylation of Cx43 S368 requires dephosphorylation of S365, as work from Solan et al. 2007, demonstrated that phosphorylation of these sites is mutually exclusive [135].

Phosphorylation of Cx43 by PKC occurs via indirect mechanisms following phosphorylation by Src [102]. Cx43 residue S368 is well established as a site for PKC phosphorylation and this site is correlated with a decrease in unitary conductance of approximately 50% (~100 pS down to ~50 pS; [136]). This decrease works together with phosphorylation by MAPK on S262 to close the channel completely. Since MAPK and PKC interact with and phosphorylate Cx43 over different regions it is likely, they can both interact simultaneously. Indeed, time course experiments following the changes in levels of site-specific phosphorylations (MAPK and PKC sites) following treatment of porcine aorta endothelial cells with vascular endothelial growth factor (VEGF) revealed a concomitant increase in phosphorylation on S255, S262, S279/282, and S368 [137]. However, the same study demonstrated that inhibition of PKC by GF109203X also resulted in a decrease in phosphorylation of S255, S279/282, and S368. The authors suggest it is likely the PKC phosphorylation may precede MAPK phosphorylation at least in VEGF activated cells to create a binding site for AP2 [137]. Similar phosphorylation patterns occur in a number of other cell types with different initiating stimuli suggesting this as a likely critical kinase program for the closure and internalization of Cx43 gap junctions [138,139,140]. Furthermore, in the same study the authors demonstrated that the phosphomimetic Cx43CT S365D mutation resulted in a significant change in structure of CT residues (T275-A276, G285-Y286, L356-S368, and R370-D379) as indicated by significant changes in chemical shift as observed in a heteronuclear single quantum coherence experiment [135]. Taken together these two lines of data suggest that phosphorylation of Cx43 by PKA on S365, induces a shift in structure which precludes binding of and phosphorylation by PKC. Finally, activation of PKC can halt the assembly of new gap junctions and its phosphorylation on S368 has been implicated in affecting gating and/or disassembly [141,142].

AP2 is one protein member of a family of five adaptor protein complexes (AP1-5) that are involved in both clathrin and non-clathrin (AP4/5) mediated trafficking events (for review see [143]). AP2 associates specifically with its cargo proteins via either two tyrosine based sorting motifs (YXXΦ or NPXY) or dileucine based sorting motifs ([D/E]XXXL[L/I]) (for review see [144]). The Cx43CT domain contains three tyrosine based sorting motifs (S1-Y^230^VFF, S2-Y^265^AYF, and S3-Y^286^KLV; [134,145]). Only S2 and S3 interacted with AP2 to initiate clathrin-mediated internalization [134]. S1 was not involved due to its membrane juxtaposition. Furthermore, the study by Thomas et al. 2003, illustrated that the Cx43 AP2 S3 overlaps with the proline rich PPXY motif which Nedd4 recognizes [145]. This suggests that it is unlikely both Nedd4 and AP2 bind Cx43 at the same time, indicating potential diverging roles for ubiquitin and clathrin mediated internalization. The significance of Cx43 containing two tyrosine based sorting signal is unclear, however, work by Johnson et al. 2013, using yeast two-hybrid analysis indicated that the Cx43CT with a Y286A mutation (abolishing S3) did not function as bait for the µ2 subunit of the AP2 complex [140]. Although they suggest a requirement for post-translational modification [140], most likely, coordination of the tyrosine ring is important for binding AP2 as tyrosine phosphorylation within the Yxxφ-type-binding motif of other proteins inhibits the interaction with AP2 (e.g., [146]).

Two additional proteins that directly interact with Cx43 are calmodulin (CaM) and CaM-dependent kinase 2 (CaMKII). Ca^2+^/CaM activates CaMKII leading to autophosphorylation and subsequent phosphorylation of target proteins, including Cx43 [147,148,149]. In vitro work using mass spectroscopy identified extensive phosphorylation of the Cx43CT by CaMKII (15 Cx43CT residues; [147]). Whether all of these sites identified occur in vivo remains to be determined as this high degree of phosphorylation could be a result of non-specific binding under in vitro conditions as the only identified CaMKII consensus is R-X-X-S/T (only four in the Cx43CT domain; for review see [150]). However, of the sites identified by Huang et al. 2011, phosphorylation of S306 has been shown to increase rather than decrease coupling [148]. NMR experiments showed that CaM directly binds the Cx43 cytoplasmic loop residues K136-S158 [151]. This occurs in a Ca^2+^ dependent manner and leads to gap junction channel closure, perhaps via occlusion of the pore (for review see [152]). We recently identified that CaM also binds Cx43CT residues K264-T290 [153]. It is tempting to speculate that this may be the mechanism by which Cx43 channels close, but remain at the plasma membrane, unlike the effects of Src phosphorylation. Along with regular turnover, gap junctions disassemble during cell division as they serve as a source of cell–cell adhesion (for review see [154]). During mitosis Cx43 phosphorylation patterns change with phosphorylation detected on S255 and S262 [155]. These changes in phosphorylation correlate with reduced intercellular communication as well as increased concentration of Cx43 in intracellular structures [156,157,158]. Interestingly, a pool of this internalized Cx43 can be recycled to nucleate the formation of new gap junction channels [155]. Similar to phosphorylation of S255 and S262 by MAPK, cyclin-dependent kinase 1 (CDK1) phosphorylates these same residues to closes the gap junction channel [156,157].

In addition to the phosphorylation-mediated changes in protein partner associations described above, new studies have begun to illustrate Cx43 as a potential target for proteolytic cleavage in various pathologies [159,160,161,162]. Lindsey et al. 2006, using in vivo, in vitro, and in silico methods demonstrated that Cx43 is a substrate for matrix metalloproteinase-7 (MMP-7; [159]). The Cx43CT domain contains two putative MMP-7 cleavage sites (G350-R362 and R374-I382); however, biochemical analysis using epitope-mapped antibodies (antibody 1: 252-270, antibody 2: 363-382) suggested cleavage was occurring only at the R374-I382 site [159]. A direct MMP-7 interaction with Cx43 was shown by SPR, in proximity to S373, suggesting potential regulation by PKA/AKT [83,88,89,159].

## 3. The Intercalated Disc as a Hub of Cx43 Mediated Protein–Protein Interactions

Cx43 is expressed in a large variety of cells [5], where it may interact with the proteins discussed above as well as yet unidentified binding partners. The expression and localization of the interacting partners vary between cell types, which possibly underlie the bewildering number of contradictory findings on the role and regulation of Cx43. In the following, we will give examples from the current knowledge about interactions and regulation of Cx43 at the intercalated disc (ID) of cardiomyocytes. The ID is a region of particular interest since it contains large amounts of Cx43 in close contact with several known interaction partners [163]. Although we only have evidence of direct interaction with a few of the nearby proteins, the list of possible partners is growing. Using a proteomics approach, Girao and coworkers showed that 236 proteins precipitated with Cx43 isolated from rat hearts [164]. Even if a lot of these are not direct or may occur outside the ID, the number of potential partners is overwhelming.

### 3.1. Nedd4 Regulates the Cx43 Content of Cardiac Gap Junctions

The ubiquitin ligase Nedd4 interacts directly with Cx43 [132] and both proteins co-localize in cardiomyocytes [165,166]. Studies indicate that multiple pathways may induce Cx43 ubiquitination in cardiomyocytes, such as activation of G-protein coupled receptors [166] and cardiac ischemia [165], and that the underlying mechanism may differ between experimental models. In the case of G-protein-coupled receptor activation, ubiquitination was achieved via a depletion of PIP2 without a measurable change in Cx43-Nedd4 co-IP [166,167], whereas cardiac ischemia increased both co-localization at the ID and increased co-IP [165]. Rather than closing the channel per se, ubiquitination most likely targets Cx43 to internalization [131] that may involve binding to the adaptor protein Eps15 [45] followed by endocytosis and lysosomal degradation [168].

### 3.2. Cx43 Regulates the Forward Trafficking of the Cardiac Sodium Channel Na_V_1.5

In contrast to the binding of Nedd4 that primarily regulates the Cx43-dependent coupling, other binding partners may be important for the regulation of nearby partners. This has proven particularly crucial at the ID, as evidenced by the fact that mutations in a number of ID components lead to wide spread dysregulation of ID function [169]. Although the exact nature of cross regulation remains obscure for many ID interactions, the interdependence of Cx43 and the cardiac sodium channel Na_V_1.5 has recently been unraveled in some detail.

Knock out of Cx43 in the heart leads to severe arrhythmias [170,171], originally believed to rely solely on the lack of intercellular coupling. However, several lines of evidence suggested a co-regulation of Cx43 and Na_V_1.5 [26,172]; and van Rijen and coworkers demonstrated that Cx43 knock out indeed reduces sodium channel expression in mice in vivo [173], a result that was reproduced in the cardiac HL-1 cell line, where Cx43 knock down reduces sodium current [173]. Intriguingly, the deletion of the last five amino acids of the Cx43CT (D378stop), which interact with the scaffolding protein ZO-1, also induced a highly arrhythmogenic phenotype in mice, despite an apparently normal intercellular coupling [174]. As for the complete loss of Cx43 described above, sodium current as well as Na_V_1.5 expression were reduced in cardiomyocytes from D378stop mice [174], showing that an intact CT is needed for full Na_V_1.5 expression at the membrane. The lack of Na_V_1.5 at the ID suggested that forward trafficking of Na_V_1.5 might be compromised. Using super resolution microscopy Agullo-Pascual et al. demonstrated that the plus end microtubule marker EB1 was partially dislocated from the ID in mice expressing Cx43-D378stop, which correlated with the presence of Na_V_1.5 clusters that came very close to the ID membrane without reaching it properly [175]. This led to the hypothesis that Cx43 acts as an anchoring point for microtubules and thereby regulates the forward trafficking of other proteins to the ID. Such an anchoring function was already demonstrated by Lo and coworkers, who showed that KO of Cx43 reduces fibroblast motility and destabilizes the microtubular network [107]. Deletion of the tubulin binding domain between amino acids 234 and 243 in the Cx43-CT recapitulated the effect of removing Cx43 altogether [107], demonstrating the important functional role of the Cx43-tubulin interaction. The role of the Cx43-tubulin interaction was also demonstrated in the cardiac HL1 cell line. As mentioned above, knock down of Cx43 in HL1 cells reduces the sodium current by ~50% and re-transfection with Cx43 restores the sodium current [173]. In contrast, transfection of the same HL1 cells with Cx43 with the tubulin binding domain truncated, failed to restore sodium current [175], supporting a role for Cx43 as a microtubule anchoring point and thereby for guiding in sodium channels. Using the HL1 cells, it was also demonstrated that Cx43-D378stop channels were unable to restore the sodium current [175], indicating that both the tubulin- and ZO-1-binding domains are needed for proper transportation of sodium channels to the membrane.

### 3.3. Cx43, the Area Composita and the Connexome

There is overwhelming evidence indicating that the functions of Cx43 extend beyond that of forming gap junction channels. Studies from various laboratories indicate that in fact, Cx43 is not only localized at the gap junction or in the perinexus [176], but also as part of a molecular/structural conglomerate named the “area composita” [177]. This term was coined to describe the fact that in the heart cells, in addition to well-defined desmosomes, there are structures with features of both, desmosomes and adherens junctions. Work of Agullo-Pascual et al. 2014 showed that Cx43 can be localized to these structures [178]. Furthermore, loss of Cx43 can decrease intercellular adhesion strength [179]. Finally, changes in desmosomal molecules can affect the integrity of gap junctions [180]. All of these complex interactions have brought us to the conclusion that in the heart, desmosomes, gap junctions, and sodium channel complexes are not separated and apart from each other. Instead, they form a protein interacting network where molecules classically defined as belonging to one of these groups, interact with others and together bring about excitability, adhesion, and intercellular coupling in the heart. This protein interacting network (dubbed “the connexome” [178,181,182]) provides for a coordinated response between the different elements that are necessary for an integrated functional syncytium.

## 4. Conclusions

It has been over 30 years since the description by Beyer, Paul, and Goodenough of Cx43 as the major gap junction protein in the heart [183]. Since this description, there has been abundant research demonstrating that Cx43 is far from a lonely and aloof piece of the intercalated disc, geared for only one function. Rather, Cx43 is part of a complex interacting protein network, not only as a recipient of interactors that modify gap junctions, but also as a component of complexes that exert other functions. As such, the view of Cx43 as a single-function molecule (to make gap junctions) is now changed to that of a multi-tasking protein, webbed into other networks to synchronize cell coupling. The extent to which those functions are involved in disease remains a matter of controversy. Whether gap junctions, or Cx43, participate in arrhythmia syndromes, or in limiting the size of infarcts, or as good (or bad?) pharmacological targets, remains incompletely defined. These last 30 years have brought us a long way in understanding Cx43 as part of a molecular ecosystem. Hopefully, the next 30 years will help us improve our ability to forecast the storms that may result from Cx43 deficiency.

## Figures and Tables

**Table 1:** Proteins suggested to interact with Cx43, but where no evidence currently exist for a direct protein–protein interaction. Abbreviations: IP, immunoprecipitation; co-Loc, co-localization; PLA, proximity ligation assay; TEM, transmission electron microscopy; PD, pull-down; IV, in vitro assay; FW, Far-Western.

Interacting Protein	Type of Detection	References
Actin	co-Loc	[21,22,23]
AGS8	IP, co-Loc	[24]
A-kinase anchoring protein 95	IP, co-Loc	[25]
Ankyrin G	IP	[26]
Apoptosis-inducing factor	IP, co-Loc, PLA	[27]
Atg16L/Atg14/Atg9/Vps34	IP, co-Loc	[28]
Bax	IP, co-Loc	[29]
β-arrestin	IP, co-Loc	[30]
β-subunit of the electron-transfer protein	IP, co-Loc, PLA	[27]
Brain-derived integrating factor-1	IP, co-Loc	[31]
CASK (LIN2)	IP, co-Loc	[32]
Caveolin-1,2,3	IP, co-Loc	[33,34,35]
Clathrin	IP, co-Loc	[36]
Claudin 5	IP, co-Loc	[37]
CIP85	IP, co-Loc	[38]
Consortin	IP, co-Loc	[39]
Cyclin E	IP, PLA, TEM	[40]
Desmocollin-2a	PD	[41]
Dlg	co-Loc	[42]
Dynamin	IP, co-Loc	[43]
EB1	IP	[44]
Eps15	IP, co-Loc	[45]
ERp29	IP, co-Loc	[46]
Hrs	co-Loc	[47]
HSP70	IP, PD	[48]
HSP90	IP, co-Loc	[49]
Light chain 3	IP, co-Loc	[50,51]
Lin-7	PD	[52]
Myosin-VI	co-Loc	[53]
DMPK	IP, co-Loc	[54]
Na_V_1.5	co-Loc	[55]
N-cadherin	co-Loc	[56]
NOV/CCN3	IP, PD	[57]
Occludin	IP, co-Loc	[37]
p120^ctn^	co-Loc	[58]
P2X7	IP, co-Loc	[59,60]
P62	IP	[50]
PKG	IV	[61]
Plakophilin-2	co-Loc	[62]
PP1/PP2A	IP, co-Loc	[63]
RPTPµ	IP	[64]
Smurf2	IP, co-Loc	[65]
STAMBP (AMSH)	IP, co-Loc	[66]
TOM20	IP, co-Loc	[49]
TRIM21	IP, co-Loc	[67]
USP8	IP	[68]
Vinculin	IP, co-Loc	[60]
Wwp1	IP	[69]
ZO-2	IP, co-Loc, PD, FW	[52,70]

## Abbreviations

AA	amino acids
AGS8	Activator of G protein signaling 8
AKT	protein kinase B
AMSH	associated molecule with the SH3 domain of STAM
AP2	adaptor protein 2
Atg	Autophagy-related protein
β-tub	β-tubulin
CaM	Calmodulin
CaMKII	Ca2+/calmodulin-dependent protein kinase II
CASK	Ca2+/calmodulin-activated serine kinase
CCN3	CYR61/CTGF/NOV
CDK1	Cyclin-dependent kinase 1
CIP75	connexin interacting protein 75 kDa
CIP85	Cx43-interacting protein of 85-kDa
CK1	Casein kinase 1
co-Loc	co-localization
CT	carboxyl terminal
Cx43	connexin 43
Cx43CT	Cx43 carboxyl terminal
Dlg	Discs-large
DMPK	dystrophia myotonica protein kinase
Drebrin	Developmentally Regulated Brain Protein 1
EB1	End binding 1
Eps15	Epidermal growth factor receptor substrate 15
ER	endoplasmic reticulum
ERp29	Endoplasmic reticulum protein 29
FERM domain	Domain found in 4.1 protein (F), Ezrin, Radixin and Moesin
FW	Far-Western
Hrs	hepatocyte growth factor-regulated tyrosine kinase substrate
HSP70	heat shock protein 70
HSP90	heat shock protein 90
ID	intercalated disc
IP	immunoprecipitation
IV	in vitro assay
Lin-7	linage-7
MAPK	Mitogen-activated protein kinase
MMP7	matrix metalloproteinase-7
Nedd4	Neural precursor cell expressed developmentally down-regulated protein 4
NMR	nuclear magnetic resonance
NOV	nephroblastoma overexpressed
p120ctn	p120-catenin
PD	pull-down
PIP2	Phosphatidylinositol-bisphosphate
PKA	protein kinase A
PKC	protein kinase C
PKG	protein kinase G
PLA	proximity ligation assay
PP	protein phosphatase
RPTPµ	receptor-like protein tyrosine phosphatase µ
SH3	Src homology 3 domain
Smurf2	Smad ubiquitination regulatory factor-2
SPR	surface plasmon resonance
STAMBP	Signal transducing adapter molecule 1 binding protein
TC-PTP	T-cell protein tyrosine phosphatase
TEM	transmission electron microscopy
TOM20	mitochondrial outer membrane receptor 20
TRIM21	Tripartite motif-containing protein 21
Tsg101	Tumor susceptibility gene 101 protein
Tyk2	Tyrosine kinase 2
UBA	Ubiquitin-associating domain
UEV	Ubiquitin E2 variant domain
USP8	Ubiquitin specific protease 8
VEGF	Vascular endothelial growth factor
ZO-1	Zonula occludens-1
ZO-2	Zonula occludens-2

## References

[1] Laird D.W. (2010). The gap junction proteome and its relationship to disease. Trends Cell Biol..

[2] Lampe P.D., Lau A.F. (2004). The effects of connexin phosphorylation on gap junctional communication. Int. J. Biochem. Cell Biol..

[3] Herve J., Bourmeyster N., Sarrouilhe D., Duffy H. (2007). Gap junctional complexes: From partners to functions. Progress. Biophys. Mol. Biol..

[4] Thevenin A.F., Kowal T.J., Fong J.T., Kells R.M., Fisher C.G., Falk M.M. (2013). Proteins and mechanisms regulating gap-junction assembly, internalization, and degradation. Physiology (Bethesda).

[5] Nielsen M.S., Axelsen L.N., Sorgen P.L., Verma V., Delmar M., Holstein-Rathlou N.H. (2012). Gap junctions. Compr. Physiol..

[6] Moreno A.P., Chanson M., Elenes S., Anumonwo J., Scerri I., Gu H., Taffet S.M., Delmar M. (2002). Role of the carboxyl terminal of connexin43 in transjunctional fast voltage gating. Circ. Res..

[7] Morley G.E., Taffet S.M., Delmar M. (1996). Intramolecular interactions mediate ph regulation of connexin43 channels. Biophys. J..

[8] Anumonwo J.M., Taffet S.M., Gu H., Chanson M., Moreno A.P., Delmar M. (2001). The carboxyl terminal domain regulates the unitary conductance and voltage dependence of connexin40 gap junction channels. Circ. Res..

[9] Revilla A., Castro C., Barrio L.C. (1999). Molecular dissection of transjunctional voltage dependence in the connexin-32 and connexin-43 junctions. Biophys. J..

[10] Sorgen P.L., Duffy H.S., Sahoo P., Coombs W., Delmar M., Spray D.C. (2004). Structural changes in the carboxyl terminus of the gap junction protein connexin43 indicates signaling between binding domains for c-src and zonula occludens-1. J. Biol. Chem..

[11] Bouvier D., Kieken F., Kellezi A., Sorgen P.L. (2008). Structural changes in the carboxyl terminus of the gap junction protein connexin 40 caused by the interaction with c-src and zonula occludens-1. Cell Commun. Adhes..

[12] Stauch K., Kieken F., Sorgen P. (2012). Characterization of the structure and intermolecular interactions between the connexin 32 carboxyl-terminal domain and the protein partners synapse-associated protein 97 and calmodulin. J. Biol. Chem..

[13] Nelson T.K., Sorgen P.L., Burt J.M. (2013). Carboxy terminus and pore-forming domain properties specific to cx37 are necessary for cx37-mediated suppression of insulinoma cell proliferation. Am. J. Physiol. Cell Physiol..

[14] Gilleron J., Carette D., Chevallier D., Segretain D., Pointis G. (2012). Molecular connexin partner remodeling orchestrates connexin traffic: From physiology to pathophysiology. Crit. Rev. Biochem. Mol. Biol..

[15] Leithe E., Mesnil M., Aasen T. (2018). The connexin 43 c-terminus: A tail of many tales. Biochim. Biophys. Acta.

[16] Solan J.L., Lampe P.D. (2018). Spatio-temporal regulation of connexin43 phosphorylation and gap junction dynamics. Biochim. Biophys. Acta.

[17] Falk M.M., Bell C.L., Kells Andrews R.M., Murray S.A. (2016). Molecular mechanisms regulating formation, trafficking and processing of annular gap junctions. BMC Cell Biol..

[18] Axelsen L.N., Calloe K., Holstein-Rathlou N.H., Nielsen M.S. (2013). Managing the complexity of communication: Regulation of gap junctions by post-translational modification. Front. Pharmacol..

[19] Xue B., Romero P.R., Noutsou M., Maurice M.M., Rudiger S.G., William A.M., Mizianty M.J., Kurgan L., Uversky V.N., Dunker A.K. (2013). Stochastic machines as a colocalization mechanism for scaffold protein function. FEBS Lett..

[20] Grosely R., Kopanic J.L., Nabors S., Kieken F., Spagnol G., Al-Mugotir M., Zach S., Sorgen P.L. (2013). Effects of phosphorylation on the structure and backbone dynamics of the intrinsically disordered connexin43 c-terminal domain. J. Biol. Chem..

[21] Yamane Y., Shiga H., Asou H., Haga H., Kawabata K., Abe K., Ito E. (1999). Dynamics of astrocyte adhesion as analyzed by a combination of atomic force microscopy and immuno-cytochemistry: The involvement of actin filaments and connexin 43 in the early stage of adhesion. Arch. Histol. Cytol..

[22] Squecco R., Sassoli C., Nuti F., Martinesi M., Chellini F., Nosi D., Zecchi-Orlandini S., Francini F., Formigli L., Meacci E. (2006). Sphingosine 1-phosphate induces myoblast differentiation through cx43 protein expression: A role for a gap junction-dependent and -independent function. Mol. Biol. Cell.

[23] Wall M.E., Otey C., Qi J., Banes A.J. (2007). Connexin 43 is localized with actin in tenocytes. Cell Motil. Cytoskelet..

[24] Sato M., Jiao Q., Honda T., Kurotani R., Toyota E., Okumura S., Takeya T., Minamisawa S., Lanier S.M., Ishikawa Y. (2009). Activator of g protein signaling 8 (ags8) is required for hypoxia-induced apoptosis of cardiomyocytes: Role of g betagamma and connexin 43 (cx43). J. Biol. Chem..

[25] Chen X., Kong X., Zhuang W., Teng B., Yu X., Hua S., Wang S., Liang F., Ma D., Zhang S. (2016). Dynamic changes in protein interaction between akap95 and cx43 during cell cycle progression of a549 cells. Sci. Rep..

[26] Sato P.Y., Coombs W., Lin X., Nekrasova O., Green K.J., Isom L.L., Taffet S.M., Delmar M. (2011). Interactions between ankyrin-g, plakophilin-2, and connexin43 at the cardiac intercalated disc. Circ. Res..

[27] Denuc A., Nunez E., Calvo E., Loureiro M., Miro-Casas E., Guaras A., Vazquez J., Garcia-Dorado D. (2016). New protein-protein interactions of mitochondrial connexin 43 in mouse heart. J. Cell. Mol. Med..

[28] Bejarano E., Yuste A., Patel B., Stout R.F., Spray D.C., Cuervo A.M. (2014). Connexins modulate autophagosome biogenesis. Nat. Cell Biol..

[29] Sun Y., Zhao X., Yao Y., Qi X., Yuan Y., Hu Y. (2012). Connexin 43 interacts with bax to regulate apoptosis of pancreatic cancer through a gap junction-independent pathway. Int. J. Oncol..

[30] Bivi N., Lezcano V., Romanello M., Bellido T., Plotkin L.I. (2011). Connexin43 interacts with betaarrestin: A pre-requisite for osteoblast survival induced by parathyroid hormone. J. Cell. Biochem..

[31] Ito T., Ueki T., Furukawa H., Sato K. (2011). The identification of novel protein, brain-derived integrating factor-1 (bdif1), which interacts with astrocytic gap junctional protein. Neurosci. Res..

[32] Marquez-Rosado L., Singh D., Rincon-Arano H., Solan J.L., Lampe P.D. (2012). Cask (lin2) interacts with cx43 in wounded skin and their coexpression affects cell migration. J. Cell Sci..

[33] Schubert A.L., Schubert W., Spray D.C., Lisanti M.P. (2002). Connexin family members target to lipid raft domains and interact with caveolin-1. Biochemistry.

[34] Langlois S., Cowan K.N., Shao Q., Cowan B.J., Laird D.W. (2008). Caveolin-1 and -2 interact with connexin43 and regulate gap junctional intercellular communication in keratinocytes. Mol. Biol. Cell.

[35] Liu L., Li Y., Lin J., Liang Q., Sheng X., Wu J., Huang R., Liu S., Li Y. (2010). Connexin43 interacts with caveolin-3 in the heart. Mol. Biol. Rep..

[36] Huang X.D., Horackova M., Pressler M.L. (1996). Changes in the expression and distribution of connexin 43 in isolated cultured adult guinea pig cardiomyocytes. Exp. Cell Res..

[37] Nagasawa K., Chiba H., Fujita H., Kojima T., Saito T., Endo T., Sawada N. (2006). Possible involvement of gap junctions in the barrier function of tight junctions of brain and lung endothelial cells. J. Cell. Physiol..

[38] Lan Z., Kurata W.E., Martyn K.D., Jin C., Lau A.F. (2005). Novel rab gap-like protein, cip85, interacts with connexin43 and induces its degradation. Biochemistry.

[39] Del Castillo F.J., Cohen-Salmon M., Charollais A., Caille D., Lampe P.D., Chavrier P., Meda P., Petit C. (2010). Consortin, a trans-golgi network cargo receptor for the plasma membrane targeting and recycling of connexins. Hum. Mol. Genet..

[40] Johnstone S.R., Kroncke B.M., Straub A.C., Best A.K., Dunn C.A., Mitchell L.A., Peskova Y., Nakamoto R.K., Koval M., Lo C.W. (2012). Mapk phosphorylation of connexin 43 promotes binding of cyclin e and smooth muscle cell proliferation. Circ. Res..

[41] Gehmlich K., Lambiase P.D., Asimaki A., Ciaccio E.J., Ehler E., Syrris P., Saffitz J.E., McKenna W.J. (2011). A novel desmocollin-2 mutation reveals insights into the molecular link between desmosomes and gap junctions. Heart Rhythm.

[42] Macdonald A.I., Sun P., Hernandez-Lopez H., Aasen T., Hodgins M.B., Edward M., Roberts S., Massimi P., Thomas M., Banks L. (2012). A functional interaction between the maguk protein hdlg and the gap junction protein connexin 43 in cervical tumour cells. Biochem. J..

[43] Gilleron J., Carette D., Fiorini C., Dompierre J., Macia E., Denizot J.P., Segretain D., Pointis G. (2011). The large gtpase dynamin2: A new player in connexin 43 gap junction endocytosis, recycling and degradation. Int. J. Biochem. Cell Biol..

[44] Shaw R.M., Fay A.J., Puthenveedu M.A., von Zastrow M., Jan Y.N., Jan L.Y. (2007). Microtubule plus-end-tracking proteins target gap junctions directly from the cell interior to adherens junctions. Cell.

[45] Girao H., Catarino S., Pereira P. (2009). Eps15 interacts with ubiquitinated cx43 and mediates its internalization. Exp. Cell Res..

[46] Das S., Smith T.D., Sarma J.D., Ritzenthaler J.D., Maza J., Kaplan B.E., Cunningham L.A., Suaud L., Hubbard M.J., Rubenstein R.C. (2009). Erp29 restricts connexin43 oligomerization in the endoplasmic reticulum. Mol. Biol. Cell.

[47] Leithe E., Kjenseth A., Sirnes S., Stenmark H., Brech A., Rivedal E. (2009). Ubiquitylation of the gap junction protein connexin-43 signals its trafficking from early endosomes to lysosomes in a process mediated by hrs and tsg101. J. Cell Sci..

[48] Hatakeyama T., Dai P., Harada Y., Hino H., Tsukahara F., Maru Y., Otsuji E., Takamatsu T. (2013). Connexin43 functions as a novel interacting partner of heat shock cognate protein 70. Sci. Rep..

[49] Rodriguez-Sinovas A., Boengler K., Cabestrero A., Gres P., Morente M., Ruiz-Meana M., Konietzka I., Miro E., Totzeck A., Heusch G. (2006). Translocation of connexin 43 to the inner mitochondrial membrane of cardiomyocytes through the heat shock protein 90-dependent tom pathway and its importance for cardioprotection. Circ. Res..

[50] Bejarano E., Girao H., Yuste A., Patel B., Marques C., Spray D.C., Pereira P., Cuervo A.M. (2012). Autophagy modulates dynamics of connexins at the plasma membrane in a ubiquitin-dependent manner. Mol. Biol. Cell.

[51] Martins-Marques T., Catarino S., Zuzarte M., Marques C., Matafome P., Pereira P., Girao H. (2015). Ischaemia-induced autophagy leads to degradation of gap junction protein connexin43 in cardiomyocytes. Biochem. J..

[52] Singh D., Lampe P.D. (2003). Identification of connexin-43 interacting proteins. Cell Commun. Adhes..

[53] Piehl M., Lehmann C., Gumpert A., Denizot J.P., Segretain D., Falk M.M. (2007). Internalization of large double-membrane intercellular vesicles by a clathrin-dependent endocytic process. Mol. Biol. Cell.

[54] Schiavon G., Furlan S., Marin O., Salvatori S. (2002). Myotonic dystrophy protein kinase of the cardiac muscle: Evaluation using an immunochemical approach. Microsc. Res. Tech..

[55] Malhotra J.D., Thyagarajan V., Chen C., Isom L.L. (2004). Tyrosine-phosphorylated and nonphosphorylated sodium channel beta1 subunits are differentially localized in cardiac myocytes. J. Biol. Chem..

[56] Akar F.G., Spragg D.D., Tunin R.S., Kass D.A., Tomaselli G.F. (2004). Mechanisms underlying conduction slowing and arrhythmogenesis in nonischemic dilated cardiomyopathy. Circ. Res..

[57] Fu C.T., Bechberger J.F., Ozog M.A., Perbal B., Naus C.C. (2004). Ccn3 (nov) interacts with connexin43 in c6 glioma cells: Possible mechanism of connexin-mediated growth suppression. J. Biol. Chem..

[58] Xu X., Li W.E., Huang G.Y., Meyer R., Chen T., Luo Y., Thomas M.P., Radice G.L., Lo C.W. (2001). Modulation of mouse neural crest cell motility by n-cadherin and connexin 43 gap junctions. J. Cell Biol..

[59] Fortes F.S., Pecora I.L., Persechini P.M., Hurtado S., Costa V., Coutinho-Silva R., Braga M.B., Silva-Filho F.C., Bisaggio R.C., De Farias F.P. (2004). Modulation of intercellular communication in macrophages: Possible interactions between gap junctions and p2 receptors. J. Cell Sci..

[60] Iacobas D.A., Suadicani S.O., Iacobas S., Chrisman C., Cohen M.A., Spray D.C., Scemes E. (2007). Gap junction and purinergic p2 receptor proteins as a functional unit: Insights from transcriptomics. J. Membr. Biol..

[61] Kwak B.R., Saez J.C., Wilders R., Chanson M., Fishman G.I., Hertzberg E.L., Spray D.C., Jongsma H.J. (1995). Effects of cgmp-dependent phosphorylation on rat and human connexin43 gap junction channels. Pflugers Arch..

[62] Li M.W., Mruk D.D., Lee W.M., Cheng C.Y. (2009). Connexin 43 and plakophilin-2 as a protein complex that regulates blood-testis barrier dynamics. Proc. Natl. Acad. Sci. USA.

[63] Ai X., Pogwizd S.M. (2005). Connexin 43 downregulation and dephosphorylation in nonischemic heart failure is associated with enhanced colocalized protein phosphatase type 2a. Circ. Res..

[64] Lezcano V., Bellido T., Plotkin L.I., Boland R., Morelli S. (2014). Osteoblastic protein tyrosine phosphatases inhibition and connexin 43 phosphorylation by alendronate. Exp. Cell Res..

[65] Fykerud T.A., Kjenseth A., Schink K.O., Sirnes S., Bruun J., Omori Y., Brech A., Rivedal E., Leithe E. (2012). Smad ubiquitination regulatory factor-2 controls gap junction intercellular communication by modulating endocytosis and degradation of connexin43. J. Cell Sci..

[66] Ribeiro-Rodrigues T.M., Catarino S., Marques C., Ferreira J.V., Martins-Marques T., Pereira P., Girao H. (2014). Amsh-mediated deubiquitination of cx43 regulates internalization and degradation of gap junctions. FASEB J..

[67] Chen V.C., Kristensen A.R., Foster L.J., Naus C.C. (2012). Association of connexin43 with e3 ubiquitin ligase trim21 reveals a mechanism for gap junction phosphodegron control. J. Proteome Res..

[68] Sun J., Hu Q., Peng H., Peng C., Zhou L., Lu J., Huang C. (2018). The ubiquitin-specific protease usp8 deubiquitinates and stabilizes cx43. J. Biol. Chem..

[69] Basheer W.A., Harris B.S., Mentrup H.L., Abreha M., Thames E.L., Lea J.B., Swing D.A., Copeland N.G., Jenkins N.A., Price R.L. (2015). Cardiomyocyte-specific overexpression of the ubiquitin ligase wwp1 contributes to reduction in connexin 43 and arrhythmogenesis. J. Mol. Cell Cardiol..

[70] Singh D., Solan J.L., Taffet S.M., Javier R., Lampe P.D. (2005). Connexin 43 interacts with zona occludens-1 and -2 proteins in a cell cycle stage-specific manner. J. Biol. Chem..

[71] Musil L.S., Goodenough D.A. (1993). Multisubunit assembly of an integral plasma membrane channel protein, gap junction connexin43, occurs after exit from the er. Cell.

[72] Kieken F., Spagnol G., Su V., Lau A.F., Sorgen P.L. (2010). Nmr structure note: Uba domain of cip75. J. Biomol. NMR.

[73] Kopanic J.L., Schlingmann B., Koval M., Lau A.F., Sorgen P.L., Su V.F. (2015). Degradation of gap junction connexins is regulated by the interaction with cx43-interacting protein of 75 kda (cip75). Biochem. J..

[74] Li X., Su V., Kurata W.E., Jin C., Lau A.F. (2008). A novel connexin43-interacting protein, cip75, which belongs to the ubl-uba protein family, regulates the turnover of connexin43. J. Biol. Chem..

[75] Su V., Hoang C., Geerts D., Lau A.F. (2014). Cip75 (connexin43-interacting protein of 75 kda) mediates the endoplasmic reticulum dislocation of connexin43. Biochem. J..

[76] Su V., Nakagawa R., Koval M., Lau A.F. (2010). Ubiquitin-independent proteasomal degradation of endoplasmic reticulum-localized connexin43 mediated by cip75. J. Biol. Chem..

[77] Thomas T., Jordan K., Simek J., Shao Q., Jedeszko C., Walton P., Laird D.W. (2005). Mechanisms of cx43 and cx26 transport to the plasma membrane and gap junction regeneration. J. Cell Sci..

[78] Akhmanova A., Steinmetz M.O. (2010). Microtubule +tips at a glance. J. Cell Sci..

[79] Welte M.A. (2004). Bidirectional transport along microtubules. Curr. Biol..

[80] Zhang S.S., Shaw R.M. (2014). Trafficking highways to the intercalated disc: New insights unlocking the specificity of connexin 43 localization. Cell Commun. Adhes..

[81] Smyth J.W., Shaw R.M. (2013). Autoregulation of connexin43 gap junction formation by internally translated isoforms. Cell Rep..

[82] Basheer W.A., Xiao S., Epifantseva I., Fu Y., Kleber A.G., Hong T., Shaw R.M. (2017). Gja1-20k arranges actin to guide cx43 delivery to cardiac intercalated discs. Circ. Res..

[83] Dukic A.R., Gerbaud P., Guibourdenche J., Thiede B., Tasken K., Pidoux G. (2017). Ezrin-anchored pka phosphorylates serine 369 and 373 on connexin 43 to enhance gap junction assembly, communication, and cell fusion. Biochem. J..

[84] Atkinson M.M., Lampe P.D., Lin H.H., Kollander R., Li X.R., Kiang D.T. (1995). Cyclic amp modifies the cellular distribution of connexin43 and induces a persistent increase in the junctional permeability of mouse mammary tumor cells. J. Cell Sci..

[85] Spray D.C., Moreno A.P., Kessler J.A., Dermietzel R. (1991). Characterization of gap junctions between cultured leptomeningeal cells. Brain Res..

[86] Pidoux G., Gerbaud P., Dompierre J., Lygren B., Solstad T., Evain-Brion D., Tasken K. (2014). A pka-ezrin-cx43 signaling complex controls gap junction communication and thereby trophoblast cell fusion. J. Cell Sci..

[87] Thevenin A.F., Margraf R.A., Fisher C.G., Kells-Andrews R.M., Falk M.M. (2017). Phosphorylation regulates connexin43/zo-1 binding and release, an important step in gap junction turnover. Mol. Biol. Cell.

[88] Dunn C.A., Lampe P.D. (2014). Injury-triggered akt phosphorylation of cx43: A zo-1-driven molecular switch that regulates gap junction size. J. Cell Sci..

[89] Dunn C.A., Su V., Lau A.F., Lampe P.D. (2012). Activation of akt, not connexin 43 protein ubiquitination, regulates gap junction stability. J. Biol. Chem..

[90] Hunter A.W., Barker R.J., Zhu C., Gourdie R.G. (2005). Zonula occludens-1 alters connexin43 gap junction size and organization by influencing channel accretion. Mol. Biol. Cell.

[91] Rhett J.M., Jourdan J., Gourdie R.G. (2011). Connexin 43 connexon to gap junction transition is regulated by zonula occludens-1. Mol. Biol. Cell.

[92] Batra N., Riquelme M.A., Burra S., Jiang J.X. (2014). 14-3-3theta facilitates plasma membrane delivery and function of mechanosensitive connexin 43 hemichannels. J. Cell Sci..

[93] Park D.J., Freitas T.A., Wallick C.J., Guyette C.V., Warn-Cramer B.J. (2006). Molecular dynamics and in vitro analysis of connexin43: A new 14-3-3 mode-1 interacting protein. Protein Sci..

[94] Park D.J., Wallick C.J., Martyn K.D., Lau A.F., Jin C., Warn-Cramer B.J. (2007). Akt phosphorylates connexin43 on ser373, a “mode-1” binding site for 14-3-3. Cell Commun. Adhes..

[95] Solan J.L., Lampe P.D. (2009). Connexin43 phosphorylation: Structural changes and biological effects. Biochem. J..

[96] Cooper C.D., Lampe P.D. (2002). Casein kinase 1 regulates connexin-43 gap junction assembly. J. Biol. Chem..

[97] Remo B.F., Qu J., Volpicelli F.M., Giovannone S., Shin D., Lader J., Liu F.Y., Zhang J., Lent D.S., Morley G.E. (2011). Phosphatase-resistant gap junctions inhibit pathological remodeling and prevent arrhythmias. Circ. Res..

[98] Giepmans B.N. (2006). Role of connexin43-interacting proteins at gap junctions. Adv. Cardiol..

[99] Giepmans B.N. (2004). Gap junctions and connexin-interacting proteins. Cardiovasc. Res..

[100] Kanemitsu M.Y., Loo L.W., Simon S., Lau A.F., Eckhart W. (1997). Tyrosine phosphorylation of connexin 43 by v-src is mediated by sh2 and sh3 domain interactions. J. Biol. Chem..

[101] Lin R., Warn-Cramer B.J., Kurata W.E., Lau A.F. (2001). V-src phosphorylation of connexin 43 on tyr247 and tyr265 disrupts gap junctional communication. J. Cell Biol..

[102] Solan J.L., Lampe P.D. (2008). Connexin 43 in la-25 cells with active v-src is phosphorylated on y247, y265, s262, s279/282, and s368 via multiple signaling pathways. Cell Commun. Adhes..

[103] Swenson K.I., Piwnica-Worms H., McNamee H., Paul D.L. (1990). Tyrosine phosphorylation of the gap junction protein connexin43 is required for the pp60v-src-induced inhibition of communication. Cell Regul..

[104] Zhou L., Kasperek E.M., Nicholson B.J. (1999). Dissection of the molecular basis of pp60(v-src) induced gating of connexin 43 gap junction channels. J. Cell Biol..

[105] Ambrosi C., Ren C., Spagnol G., Cavin G., Cone A., Grintsevich E.E., Sosinsky G.E., Sorgen P.L. (2016). Connexin43 forms supramolecular complexes through non-overlapping binding sites for drebrin, tubulin, and zo-1. PLoS ONE.

[106] Zheng Li., Li H., Spagnol G., Patel K., Sorgen P.L. Src phosphorylation of Cx43 residue Y313 contributes to inhibiting the interaction with Drebrin and gap junction disassembly. J. Mol. Cell. Cardiol..

[107] Francis R., Xu X., Park H., Wei C.J., Chang S., Chatterjee B., Lo C. (2011). Connexin43 modulates cell polarity and directional cell migration by regulating microtubule dynamics. PLoS ONE.

[108] Li H., Spagnol G., Naslavsky N., Caplan S., Sorgen P.L. (2014). Tc-ptp directly interacts with connexin43 to regulate gap junction intercellular communication. J. Cell Sci..

[109] Ai Z., Fischer A., Spray D.C., Brown A.M., Fishman G.I. (2000). Wnt-1 regulation of connexin43 in cardiac myocytes. J. Clin. Investig..

[110] Nakashima T., Ohkusa T., Okamoto Y., Yoshida M., Lee J.K., Mizukami Y., Yano M. (2014). Rapid electrical stimulation causes alterations in cardiac intercellular junction proteins of cardiomyocytes. Am. J. Physiol. Heart Circ. Physiol..

[111] Swope D., Cheng L., Gao E., Li J., Radice G.L. (2012). Loss of cadherin-binding proteins beta-catenin and plakoglobin in the heart leads to gap junction remodeling and arrhythmogenesis. Mol. Cell. Biol..

[112] Wang H.X., Gillio-Meina C., Chen S., Gong X.Q., Li T.Y., Bai D., Kidder G.M. (2013). The canonical wnt2 pathway and fsh interact to regulate gap junction assembly in mouse granulosa cells. Biol. Reprod..

[113] Rinaldi F., Hartfield E.M., Crompton L.A., Badger J.L., Glover C.P., Kelly C.M., Rosser A.E., Uney J.B., Caldwell M.A. (2014). Cross-regulation of connexin43 and beta-catenin influences differentiation of human neural progenitor cells. Cell Death Dis..

[114] Spagnol G., Trease A.J., Zheng Li., Phillips A., Sorgen P.L. Regulation of Connexin43 by the direct interaction with β-catenin. Int. J. Mol. Sci..

[115] Giepmans B.N., Hengeveld T., Postma F.R., Moolenaar W.H. (2001). Interaction of c-src with gap junction protein connexin-43. Role in the regulation of cell-cell communication. J. Biol. Chem..

[116] Gilleron J., Fiorini C., Carette D., Avondet C., Falk M.M., Segretain D., Pointis G. (2008). Molecular reorganization of cx43, zo-1 and src complexes during the endocytosis of gap junction plaques in response to a non-genomic carcinogen. J. Cell Sci..

[117] Mitra S.S., Xu J., Nicholson B.J. (2012). Coregulation of multiple signaling mechanisms in pp60v-src-induced closure of cx43 gap junction channels. J. Membr. Biol..

[118] Pahujaa M., Anikin M., Goldberg G.S. (2007). Phosphorylation of connexin43 induced by src: Regulation of gap junctional communication between transformed cells. Exp. Cell Res..

[119] Toyofuku T., Akamatsu Y., Zhang H., Kuzuya T., Tada M., Hori M. (2001). C-src regulates the interaction between connexin-43 and zo-1 in cardiac myocytes. J. Biol. Chem..

[120] Homma N., Alvarado J.L., Coombs W., Stergiopoulos K., Taffet S.M., Lau A.F., Delmar M. (1998). A particle-receptor model for the insulin-induced closure of connexin43 channels. Circ. Res..

[121] Cottrell G.T., Lin R., Warn-Cramer B.J., Lau A.F., Burt J.M. (2003). Mechanism of v-src- and mitogen-activated protein kinase-induced reduction of gap junction communication. Am. J. Physiol. Cell Physiol..

[122] Saidi Brikci-Nigassa A., Clement M.J., Ha-Duong T., Adjadj E., Ziani L., Pastre D., Curmi P.A., Savarin P. (2012). Phosphorylation controls the interaction of the connexin43 c-terminal domain with tubulin and microtubules. Biochemistry.

[123] Butkevich E., Hulsmann S., Wenzel D., Shirao T., Duden R., Majoul I. (2004). Drebrin is a novel connexin-43 binding partner that links gap junctions to the submembrane cytoskeleton. Curr. Biol..

[124] Kieken F., Mutsaers N., Dolmatova E., Virgil K., Wit A.L., Kellezi A., Hirst-Jensen B.J., Duffy H.S., Sorgen P.L. (2009). Structural and molecular mechanisms of gap junction remodeling in epicardial border zone myocytes following myocardial infarction. Circ. Res..

[125] Li H., Spagnol G., Zheng L., Stauch K.L., Sorgen P.L. (2016). Regulation of connexin43 function and expression by tyrosine kinase 2. J. Biol. Chem..

[126] Yamaoka K., Saharinen P., Pesu M., Holt V.E., Silvennoinen O., O’Shea J.J. (2004). The janus kinases (jaks). Genome Biol..

[127] Girault J.A., Labesse G., Mornon J.P., Callebaut I. (1999). The n-termini of fak and jaks contain divergent band 4.1 domains. Trends Biochem. Sci..

[128] Haan C., Kreis S., Margue C., Behrmann I. (2006). Jaks and cytokine receptors—An intimate relationship. Biochem. Pharmacol..

[129] Wilks A.F., Harpur A.G., Kurban R.R., Ralph S.J., Zurcher G., Ziemiecki A. (1991). Two novel protein-tyrosine kinases, each with a second phosphotransferase-related catalytic domain, define a new class of protein kinase. Mol. Cell Biol..

[130] Huibregtse J.M., Scheffner M., Beaudenon S., Howley P.M. (1995). A family of proteins structurally and functionally related to the e6-ap ubiquitin-protein ligase. Proc. Natl. Acad. Sci. USA.

[131] Leykauf K., Salek M., Bomke J., Frech M., Lehmann W.D., Durst M., Alonso A. (2006). Ubiquitin protein ligase nedd4 binds to connexin43 by a phosphorylation-modulated process. J. Cell Sci..

[132] Spagnol G., Kieken F., Kopanic J.L., Li H., Zach S., Stauch K.L., Grosely R., Sorgen P.L. (2016). Structural studies of the nedd4 ww domains and their selectivity for the connexin43 (cx43) carboxyl terminus. J. Biol. Chem..

[133] Auth T., Schluter S., Urschel S., Kussmann P., Sonntag S., Hoher T., Kreuzberg M.M., Dobrowolski R., Willecke K. (2009). The tsg101 protein binds to connexins and is involved in connexin degradation. Exp. Cell Res..

[134] Fong J.T., Kells R.M., Falk M.M. (2013). Two tyrosine-based sorting signals in the cx43 c-terminus cooperate to mediate gap junction endocytosis. Mol. Biol. Cell.

[135] Solan J.L., Marquez-Rosado L., Sorgen P.L., Thornton P.J., Gafken P.R., Lampe P.D. (2007). Phosphorylation at s365 is a gatekeeper event that changes the structure of cx43 and prevents down-regulation by pkc. J. Cell Biol..

[136] Ek-Vitorin J.F., King T.J., Heyman N.S., Lampe P.D., Burt J.M. (2006). Selectivity of connexin 43 channels is regulated through protein kinase c-dependent phosphorylation. Circ. Res..

[137] Nimlamool W., Andrews R.M., Falk M.M. (2015). Connexin43 phosphorylation by pkc and mapk signals vegf-mediated gap junction internalization. Mol. Biol. Cell.

[138] Cone A.C., Cavin G., Ambrosi C., Hakozaki H., Wu-Zhang A.X., Kunkel M.T., Newton A.C., Sosinsky G.E. (2014). Protein kinase cdelta-mediated phosphorylation of connexin43 gap junction channels causes movement within gap junctions followed by vesicle internalization and protein degradation. J. Biol. Chem..

[139] Fong J.T., Nimlamool W., Falk M.M. (2014). Egf induces efficient cx43 gap junction endocytosis in mouse embryonic stem cell colonies via phosphorylation of ser262, ser279/282, and ser368. FEBS Lett..

[140] Johnson K.E., Mitra S., Katoch P., Kelsey L.S., Johnson K.R., Mehta P.P. (2013). Phosphorylation on ser-279 and ser-282 of connexin43 regulates endocytosis and gap junction assembly in pancreatic cancer cells. Mol. Biol. Cell.

[141] Lampe P.D. (1994). Analyzing phorbol ester effects on gap junctional communication: A dramatic inhibition of assembly. J. Cell Biol..

[142] Lampe P.D., TenBroek E.M., Burt J.M., Kurata W.E., Johnson R.G., Lau A.F. (2000). Phosphorylation of connexin43 on serine368 by protein kinase c regulates gap junctional communication. J. Cell Biol..

[143] Kirchhausen T., Owen D., Harrison S.C. (2014). Molecular structure, function, and dynamics of clathrin-mediated membrane traffic. Cold Spring Harb. Perspect. Biol..

[144] Bonifacino J.S., Traub L.M. (2003). Signals for sorting of transmembrane proteins to endosomes and lysosomes. Annu. Rev. Biochem..

[145] Thomas M.A., Zosso N., Scerri I., Demaurex N., Chanson M., Staub O. (2003). A tyrosine-based sorting signal is involved in connexin43 stability and gap junction turnover. J. Cell Sci..

[146] Kittler J.T., Chen G., Kukhtina V., Vahedi-Faridi A., Gu Z., Tretter V., Smith K.R., McAinsh K., Arancibia-Carcamo I.L., Saenger W. (2008). Regulation of synaptic inhibition by phospho-dependent binding of the ap2 complex to a yecl motif in the gabaa receptor gamma2 subunit. Proc. Natl. Acad. Sci. USA.

[147] Huang R.Y., Laing J.G., Kanter E.M., Berthoud V.M., Bao M., Rohrs H.W., Townsend R.R., Yamada K.A. (2011). Identification of camkii phosphorylation sites in connexin43 by high-resolution mass spectrometry. J. Proteome Res..

[148] Procida K., Jorgensen L., Schmitt N., Delmar M., Taffet S.M., Holstein-Rathlou N.H., Nielsen M.S., Braunstein T.H. (2009). Phosphorylation of connexin43 on serine 306 regulates electrical coupling. Heart Rhythm.

[149] Shifman J.M., Choi M.H., Mihalas S., Mayo S.L., Kennedy M.B. (2006). Ca2+/calmodulin-dependent protein kinase ii (camkii) is activated by calmodulin with two bound calciums. Proc. Natl. Acad. Sci. USA.

[150] Braun A.P., Schulman H. (1995). The multifunctional calcium/calmodulin-dependent protein kinase: From form to function. Annu. Rev. Physiol..

[151] Zhou Y., Yang W., Lurtz M.M., Ye Y., Huang Y., Lee H.W., Chen Y., Louis C.F., Yang J.J. (2007). Identification of the calmodulin binding domain of connexin 43. J. Biol. Chem..

[152] Zou J., Salarian M., Chen Y., Veenstra R., Louis C.F., Yang J.J. (2014). Gap junction regulation by calmodulin. FEBS Lett..

[153] Spagnol G., Chenavas S., Trease A., Li H., Kieken F., Brownell S., Sorgen P.L. Characterizing the interaction between calmodulin and the Cx43 cytoplasmic domains (manuscript in preparation).

[154] Dbouk H.A., Mroue R.M., El-Sabban M.E., Talhouk R.S. (2009). Connexins: A myriad of functions extending beyond assembly of gap junction channels. Cell Commun. Signal..

[155] Boassa D., Solan J.L., Papas A., Thornton P., Lampe P.D., Sosinsky G.E. (2010). Trafficking and recycling of the connexin43 gap junction protein during mitosis. Traffic.

[156] Kanemitsu M.Y., Jiang W., Eckhart W. (1998). Cdc2-mediated phosphorylation of the gap junction protein, connexin43, during mitosis. Cell Growth Differ..

[157] Lampe P.D., Kurata W.E., Warn-Cramer B.J., Lau A.F. (1998). Formation of a distinct connexin43 phosphoisoform in mitotic cells is dependent upon p34cdc2 kinase. J. Cell Sci..

[158] Stein L.S., Boonstra J., Burghardt R.C. (1992). Reduced cell-cell communication between mitotic and nonmitotic coupled cells. Exp. Cell Res..

[159] Lindsey M.L., Escobar G.P., Mukherjee R., Goshorn D.K., Sheats N.J., Bruce J.A., Mains I.M., Hendrick J.K., Hewett K.W., Gourdie R.G. (2006). Matrix metalloproteinase-7 affects connexin-43 levels, electrical conduction, and survival after myocardial infarction. Circulation.

[160] Kowluru R.A., Mohammad G., dos Santos J.M., Zhong Q. (2011). Abrogation of mmp-9 gene protects against the development of retinopathy in diabetic mice by preventing mitochondrial damage. Diabetes.

[161] Mohammad G., Kowluru R.A. (2011). Novel role of mitochondrial matrix metalloproteinase-2 in the development of diabetic retinopathy. Invest. Ophthalmol. Vis. Sci..

[162] Wu X., Huang W., Luo G., Alain L.A. (2013). Hypoxia induces connexin 43 dysregulation by modulating matrix metalloproteinases via mapk signaling. Mol. Cell Biochem..

[163] Vermij S.H., Abriel H., van Veen T.A. (2017). Refining the molecular organization of the cardiac intercalated disc. Cardiovasc. Res..

[164] Martins-Marques T., Anjo S.I., Pereira P., Manadas B., Girao H. (2015). Interacting network of the gap junction (gj) protein connexin43 (cx43) is modulated by ischemia and reperfusion in the heart. Mol. Cell Proteom..

[165] Martins-Marques T., Catarino S., Marques C., Matafome P., Ribeiro-Rodrigues T., Baptista R., Pereira P., Girao H. (2015). Heart ischemia results in connexin43 ubiquitination localized at the intercalated discs. Biochimie.

[166] Mollerup S., Hofgaard J.P., Braunstein T.H., Kjenseth A., Leithe E., Rivedal E., Holstein-Rathlou N.H., Nielsen M.S. (2011). Norepinephrine inhibits intercellular coupling in rat cardiomyocytes by ubiquitination of connexin43 gap junctions. Cell Commun. Adhes..

[167] Hofgaard J.P., Banach K., Mollerup S., Jorgensen H.K., Olesen S.P., Holstein-Rathlou N.H., Nielsen M.S. (2008). Phosphatidylinositol-bisphosphate regulates intercellular coupling in cardiac myocytes. Pflugers Arch..

[168] Totland M.Z., Bergsland C.H., Fykerud T.A., Knudsen L.M., Rasmussen N.L., Eide P.W., Yohannes Z., Sorensen V., Brech A., Lothe R.A. (2017). The e3 ubiquitin ligase nedd4 induces endocytosis and lysosomal sorting of connexin 43 to promote loss of gap junctions. J. Cell Sci..

[169] Stroemlund L.W., Jensen C.F., Qvortrup K., Delmar M., Nielsen M.S. (2015). Gap junctions-guards of excitability. Biochem. Soc. Trans..

[170] Gutstein D.E., Morley G.E., Tamaddon H., Vaidya D., Schneider M.D., Chen J., Chien K.R., Stuhlmann H., Fishman G.I. (2001). Conduction slowing and sudden arrhythmic death in mice with cardiac-restricted inactivation of connexin43. Circ. Res..

[171] Van Rijen H.V., Eckardt D., Degen J., Theis M., Ott T., Willecke K., Jongsma H.J., Opthof T., de Bakker J.M. (2004). Slow conduction and enhanced anisotropy increase the propensity for ventricular tachyarrhythmias in adult mice with induced deletion of connexin43. Circulation.

[172] Desplantez T., McCain M.L., Beauchamp P., Rigoli G., Rothen-Rutishauser B., Parker K.K., Kleber A.G. (2012). Connexin43 ablation in foetal atrial myocytes decreases electrical coupling, partner connexins, and sodium current. Cardiovasc. Res..

[173] Jansen J.A., Noorman M., Musa H., Stein M., de Jong S., van der Nagel R., Hund T.J., Mohler P.J., Vos M.A., van Veen T.A. (2012). Reduced heterogeneous expression of cx43 results in decreased Na_V_1.5 expression and reduced sodium current that accounts for arrhythmia vulnerability in conditional cx43 knockout mice. Heart Rhythm.

[174] Lubkemeier I., Requardt R.P., Lin X., Sasse P., Andrie R., Schrickel J.W., Chkourko H., Bukauskas F.F., Kim J.S., Frank M. (2013). Deletion of the last five c-terminal amino acid residues of connexin43 leads to lethal ventricular arrhythmias in mice without affecting coupling via gap junction channels. Basic Res. Cardiol..

[175] Agullo-Pascual E., Lin X., Leo-Macias A., Zhang M., Liang F.X., Li Z., Pfenniger A., Lubkemeier I., Keegan S., Fenyo D. (2014). Super-resolution imaging reveals that loss of the c-terminus of connexin43 limits microtubule plus-end capture and Na_V_1.5 localization at the intercalated disc. Cardiovasc. Res..

[176] Rhett J.M., Gourdie R.G. (2012). The perinexus: A new feature of cx43 gap junction organization. Heart Rhythm.

[177] Franke W.W., Borrmann C.M., Grund C., Pieperhoff S. (2006). The area composita of adhering junctions connecting heart muscle cells of vertebrates. I. Molecular definition in intercalated disks of cardiomyocytes by immunoelectron microscopy of desmosomal proteins. Eur. J. Cell Biol..

[178] Agullo-Pascual E., Reid D.A., Keegan S., Sidhu M., Fenyo D., Rothenberg E., Delmar M. (2013). Super-resolution fluorescence microscopy of the cardiac connexome reveals plakophilin-2 inside the connexin43 plaque. Cardiovasc. Res..

[179] Agullo-Pascual E., Delmar M. (2012). The noncanonical functions of cx43 in the heart. J. Membr. Biol..

[180] Oxford E.M., Musa H., Maass K., Coombs W., Taffet S.M., Delmar M. (2007). Connexin43 remodeling caused by inhibition of plakophilin-2 expression in cardiac cells. Circ. Res..

[181] Agullo-Pascual E., Cerrone M., Delmar M. (2014). Arrhythmogenic cardiomyopathy and brugada syndrome: Diseases of the connexome. FEBS Lett..

[182] Leo-Macias A., Agullo-Pascual E., Delmar M. (2016). The cardiac connexome: Non-canonical functions of connexin43 and their role in cardiac arrhythmias. Semin. Cell Dev. Biol..

[183] Beyer E.C., Paul D.L., Goodenough D.A. (1987). Connexin43: A protein from rat heart homologous to a gap junction protein from liver. J. Cell Biol..

